# LMOD3: the “missing link” in nemaline myopathy?

**DOI:** 10.18632/oncotarget.5267

**Published:** 2015-08-26

**Authors:** Sarah A. Sandaradura, Kathryn N. North

**Affiliations:** Murdoch Children’s Research Institute, The Royal Children’s Hospital and Department of Paediatrics, University of Melbourne, Victoria, Australia

**Keywords:** nemaline myopathy, congenital myopathy, muscle, genetics

Understanding of disease pathogenesis and the development of effective therapies for inherited muscle disorders requires identification of the genes responsible and the role of the associated proteins within skeletal muscle.

Nemaline myopathy (NM) is one of the most common forms of congenital-onset myopathy and provides an excellent example of how mutations in many skeletal muscle genes can lead to a common clinical and pathological phenotype. NM is characterized by non-progressive muscle weakness and hypotonia and the finding of thread-like electron-dense protein accumulations or “rods” in skeletal myofibers. It encompasses a spectrum of clinical severity ranging from infants presenting in the neonatal period with profound weakness, hypotonia and respiratory insufficiency, through to adults with relatively mild weakness.

There is significant genetic heterogeneity in NM, and it can occur as an autosomal dominant (AD), sporadic (new dominant) or autosomal recessive (AR) disorder. The first six genes identified as causes of NM encode components of the sarcomeric thin filament in skeletal muscle (*NEB*, *ACTA1*, *TPM3*, *TPM2*, *CFL2* and *TNNT1*). Thus NM came to be regarded as a “disorder of the thin filament”. More recently, mutations in three genes encoding members of the BTB superfamily of proteins (*KBTBD13*, *KLHL40* and *KLHL41*) have been shown to cause NM. These “kelch” proteins are thought to be involved in transcriptional regulation, ubiquitination and protein degradation, however their role in the disease pathogenesis of NM was not well understood.

We recently identified *LMOD3*, which encodes leiomodin-3 (LMOD3), as a new cause of autosomal recessive nemaline myopathy [1]. With international collaborators we identified 21 patients with *LMOD3-NM* and showed that it typically presents with severe muscle weakness associated with marked disorganization of the sarcomere, that is usually lethal at birth or in the first few months of life. LMOD3 protein is absent in the majority of patients; in one family only, there was expression of truncated LMOD3, resulting in a less severe phenotype compatible with survival.

The leiomodin proteins (LMOD1, LMOD2 and LMOD3) are members of the tropomodulin protein family, a group of proteins that cap actin filaments, regulating actin thin filament lengths - consistent with the concept that NM is primarily due to dysregulation of turnover of the thin filament. Our recent work published in the Journal of Clinical Investigation showed that LMOD3 is expressed in skeletal muscle from the early stages of differentiation (consistent with the early onset of disease *in utero*), localizes to actin thin filaments and is a strong nucleator of the actin thin filament [1]. A zebrafish model of *LMOD3*-NM replicated the human phenotype. Muscle from patients and the zebrafish model demonstrated marked variation in (the usually very uniform) thin filament length, suggesting that LMOD3 is important for sarcomeric thin filament organization and regulation of thin filament length in skeletal muscle.

Several additional recent studies suggest that LMOD3 plays a key role in the pathogenesis of some other forms of NM and provides insight into the previously “missing link” between the thin filament structural proteins and the kelch family of proteins. Variants in *KLHL40* are associated with a severe form of NM characterized by AR inheritance [2]. Garg et al showed that KLHL40 binds to both NEB and LMOD3, and promotes the stability of these proteins [3]. LMOD3 and NEB protein levels are markedly reduced in a mouse model of *KLHL40*-NM and in patients with *KLHL40*-NM, suggesting that KLHL40 regulates LMOD3 expression and that loss of LMOD3 contributes to the muscle weakness in *KLHL40*-NM [3].

Subsequently, two KO mouse models of *LMOD3-* NM have been published and both recapitulate the severe muscle weakness, failure to thrive, muscle fiber atrophy, loss of sarcomeric organization and presence of nemaline rods, seen in humans [4, 5].

Actin expression in muscle is tightly controlled by a regulatory circuit, which involves expression of transcription factors and co-activation of additional genes encoding components of the sarcomere and cytoskeleton. Cenik et al showed that Lmod3 transcription in skeletal muscle is regulated by upstream transcription factors SRF and MEF2 and that Lmod3 enhances activity of SRF and MRTF, which, in turn, sustain Lmod3 expression [4]. This suggests that muscle weakness and sarcomeric dysfunction in *LMOD3*-NM may occur due to accumulation of G-actin monomers causing disruption of sarcomeric structure and reduced expression of transcription factors, resulting in downregulation of expression of genes encoding cytoskeletal proteins and components of the contractile apparatus [4]. Mutations in *KLHL40* associated with NM lead to a secondary loss of LMOD3 and likely trigger a similar disruption in the development and maintenance of the actin thin filament.

Intriguingly, a recent study in the frog (*Xenopus*) demonstrated redundancy of function between Lmod3 and Tmod4 (the predominant isoform of tropomodulin in mammalian skeletal muscle), suggesting a potential target for future therapies. Knockdown of either Lmod3 or Tmod4 during development resulted in disruption of sarcomeric assembly and reduction in *Xenopus* embryo motility [6]. Surprisingly, supplementation of Lmod3 deficient *Xenopus* embryos with Tmod4 (and vice versa) led to rescue of the phenotype [6].

In combination, these studies confirm the crucial role of LMOD3 in development and maintenance of sarcomeric structure and function. The identification of LMOD3 as a binding partner of KLHL40(3), the functional redundancy of Lmod3 and Tmod4 in myofibrillogenesis [6] and the identification of the “feed-forward” circuit by which LMOD3 expression influences key transcription factors in skeletal muscle [4] provide important insights into the molecular basis of muscle weakness in *LMOD3-*NM and potentially in the other genetic forms of NM. Future studies in mouse, zebrafish and human “models” of NM will explore if and how all ten (currently known) genetic forms of NM converge into a common pathogenic pathway and how we can best target this pathway to slow, reverse or prevent muscle weakness in this group of disorders.
